# Development and validation of the Chinese version of the evidence-based practice profile questionnaire (EBP^2^Q)

**DOI:** 10.1186/s12909-020-02189-z

**Published:** 2020-08-24

**Authors:** Ming-Yu Hu, Yan-Ni Wu, Maureen Patricia McEvoy, Yan-Fang Wang, Wei-Lian Cong, Li-Ping Liu, Xiao-Xia Li, Chun-Lan Zhou

**Affiliations:** 1 Department of Nursing, Nanfang Hospital, Southern Medical University, No.1838 Guangzhou Avenue North, Guangzhou, 510515 Guangdong China; 2 Allied Health and Human Performance Unit, University of South Australia, North Terrace, Adelaide, 5000 Australia

**Keywords:** Evidence-based practice, Nurse, Instrument, Education, Validation

## Abstract

**Background:**

Evidence-based practice (EBP) education or training are considered fundamental to building and strengthening an EBP culture, as well as to encouraging evidence-based academic and clinical practice in the nursing community. However, few valid and reliable instruments are available for the assessment of EBP teaching and learning in clinical nurses in China. Translation, reliability, and validity testing of the English Evidence-Based Practice Profile Questionnaire (EBP^2^Q), which has strong psychometric properties, may encourage evaluation and promote the implementation of EBP in Mainland China.

**Methods:**

Based on established guidelines for the development of questionnaires, the English EBP^2^Q was translated and cross-culturally adapted. The Chinese version of the EBP^2^Q (EBP^2^Q-C) was validated using a sample of 543 nurses. Structural validity was evaluated through exploratory factor analysis and confirmatory factor analysis, and the questionnaire was tested for convergent and criterion validity. The internal consistency and test-retest reliability were also evaluated.

**Results:**

The content validity index demonstrated good content validity (≥0.98). An eight-factor structure was obtained in the exploratory factor analysis, and verified by a three-order factor model from the confirmatory factor analysis (*χ*^*2*^*/df* = 2.001; RMSEA = 0.065; SRMR = 0.077; and CFI = 0.884). The Spearman’s rank correlation analysis of the EBP^2^Q-C with the Evidence-Based Practice Questionnaire showed moderate correlations for *Practice* (0.58) and *Confidence* (0.68) and a low correlation for *Sympathy* (0.32). Criterion validity was demonstrated by significant differences in terms of nurses’ highest education, present position, EBP training, involvement in research programs, and level of understanding of English. Both the overall Cronbach’s α and the Cronbach’s α for the domains exceeded 0.70. The intraclass correlation coefficients for the domains ranged between 0.75 and 0.96, indicating satisfactory repeatability.

**Conclusions:**

Except for the convergent validity of the *Sympathy* domain, the EBP^2^Q-C provided evidence of validity and reliability. Therefore, it can be applied in EBP education or training assessment in Mainland China.

## Background

Evidence-based practice (EBP) is an effective strategy to make conscientious and judicious clinical decisions based on the best evidence available, along with the complex clinical circumstances, clinician expertise, and patient preferences [1]. The EBP approach has contributed to better clinical outcomes, reduced unnecessary healthcare costs, and improved patient satisfaction [2, 3]. Hence, the importance of EBP in the global healthcare system is well-established [4, 5]. The International Council of Nurses has identified EBP as the gold standard for high-quality healthcare [5–7]. As one of the largest groups of healthcare workers, there is an increasing demand for nurses, to incorporate the best evidence into clinical decision making. This will assist institutions to achieve valued and cost-efficient evidence-based health care [8]. However, studies indicate that both nursing students and practicing nurses are still not ready for and competent in EBP [9, 10]. This may be due to a lack of understanding of EBP and/or to not knowing how to apply the research evidence into practical patient care [4, 10].

Education and training in EBP are reported as basic and essential approaches to enhance EBP [9, 11, 12]. The international Sicily Statement had put forward an EBP curriculum framework to facilitate EBP education and training programs [13], and research training, EBP skills, and knowledge have been embedded in nursing education [12]. Melnyk et al. [8] have also published 13 EBP competencies for clinical nurses and a further 11 for advanced nurses. Currently, nursing faculties and administrators are faced with the important challenge of exploring new and efficient instructional and training modes to build, strengthen, and encourage an EBP culture in academic and clinical practice in the nursing community [10, 14].

Psychometrically robust assessment tools are required to ascertain the efficiency of various teaching and training methods, as well as to monitor the progress of the participants in EBP [15–17]. Shaneyfelt et al. [18] analyzed 104 instruments from numerous EBP education programs across the health professionals and reported that rigorously-developed tools were in the minority, with only 10% having established validity in ≥3 areas. Furthermore, there is a limited number of EBP self-report knowledge, attitudes, and behavior questionnaires available for use with nurses. Of those that exist, many exclude coverage of the behavior domain of EBP [19–23] or lack development rigor in terms of reliability and validity [24–26]. The insufficiency of psychometric properties in EBP questionnaires for nurses was also acknowledged by Leung et al. [27].

The Evidence-Based Practice Profile Questionnaire (EBP^2^Q) developed by McEvoy et al. in Australia [28], is one of the most comprehensive self-report questionnaires that assesses the EBP knowledge, skills, attitudes, and behaviors across all five steps of EBP (Ask, Acquire, Appraise, Apply, Assess). Validated on 631 participants including students, academics, and practitioners from different healthcare professions [28], the EBP^2^Q has shown acceptable validity and reliability properties.

Panczyk et al. [29] adapted and validated the EBP^2^Q in 1362 nurses, nursing students, and midwives in Poland. Similar to the English version of the EBP^2^Q, the Polish version has shown high internal consistencies (Cronbach’s α = 0.80–0.97) and theoretical and criterion validity were confirmed [29]. The EBP^2^Q was also translated into Norwegian by Titlestad et al. [30] and validated in 149 nursing students, social education students, and health and social workers. The Norwegian EBP^2^Q showed high internal consistencies (Cronbach’s α ≥ 0.90), except for *Sympathy* (Cronbach’s α = 0.66), and demonstrated criterion and responsive validity [30].

This study was designed to translate, adapt, and validate the EBP^2^Q for use with clinical nurses in China to evaluate the self-perception of nurses on their EBP competencies, to promote the application of EBP in clinical nursing practice, and to help managers to identify where the improvement of EBP is needed.

## Methods

A validation and reliability study was undertaken at Nanfang Hospital, Southern Medical University (Guangzhou, China). This study was conducted in two stages, with stage 1 comprising the translation and adaptation of the EBP^2^Q into the Chinese version, and stage 2 evaluating the psychometric properties of the Chinese version of the EBP^2^Q. All study processes were conducted according to the Declaration of Helsinki and ethical approval for the study was obtained retrospectively on September 24th, 2019 from the Medical Ethics Committee of Nanfang Hospital of Southern Medical University (NFEC-2019-171).

### Instruments

#### Nurse general information questionnaire

Demographic characteristics and EBP-related data were collected using a questionnaire designed by the research team. The characteristics included sex, age, years of working, present position, highest education, EBP training, level of English and whether they had conducted a research study.

#### Evidence-based practice questionnaire (EBPQ)

The 24-item EBPQ was initially designed to evaluate EBP uptake and implementation of the nurses [25]. It comprises the three domains *Practice, Attitude, and Knowledge*, which are scored on seven-point Likert scales (1–7), with higher scores indicating more favorable outcomes. The EBPQ, validated on 751 clinical nurses, has good internal consistencies for the overall questionnaire (Cronbach’s α = 0.87) and the domains of *Practice, Attitudes*, and *Knowledge* (Cronbach’s α = 0.85, 0.79, and 0.91) [25]. The Chinese version of the EBPQ, as part of a Master’s thesis, was validated in 1621 clinical nursing staff with a Cronbach’s α of 0.94 for the overall questionnaire [31]. The Chinese EBPQ was also used in a multiple center cross-sectional study of 648 Chinese registered nurses [32].

#### EBP^2^Q

The EBP^2^Q incorporates 58 items in five domains: *Relevance* (14 items), relating to an individual’s perspectives on the importance of EBP; *Sympathy* (seven items), relating to the perceived compatibility of EBP with the practicality of use in day-to-day work; *Terminology* (17 items), relating to the understanding of research terms; *Practice* (nine items), relating to the application of EBP in clinical circumstances; and *Confidence* (11 items), relating to the individual’s perception of their EBP skills [28]. There are also 16 additional non-domain items related to EBP, which have not been classified into any of the known domains and were not included in the following analysis. Each of the items is rated on a five-point Likert scale from 1 = “not at all true” to 5 = “very true” in *Relevance*, from 1 = “strongly disagree” to 5 = “strongly agree” in *Sympathy*, from 1 = “never heard the term” to 5 = “understand and could explain to others” in *Terminology*, from 1 = “never” to 5 = “daily” in *Practice*, from 1 = “not at all confident” to 5 = “very confident” in *Confidence*. The seven items in *Sympathy* are negatively worded (e.g. ‘EBP does not take into account the limitations of my day-to-day work) and need to be reverse scored. Higher scores indicate more competent respondents in that particular EBP domain [28].

The EBP^2^Q has demonstrated good psychometric properties, including an excellent overall Cronbach’s α (0.96), domain intraclass correlation coefficients (ICCs) ranging between 0.77 and 0.94, and confirmed convergent validity with the EBPQ regarding the three comparable domains (with the following Pearson’s correlations: *Confidence,* 0.66; *Practice,* 0.80; and *Sympathy,* 0.54). As indicators of criterion validity, the domains *Relevance* and *Terminology* (no training vs. ≤20 h of training. no training vs. > 20 h of training) and *Confidence* (no training vs. ≤20 h of training) significantly distinguished participants by exposure to EBP training [28].

### Methods in stage 1: translation and adaptation of the EBP^2^Q

#### Procedure

##### Translation

Permission for translation and adaptation in Chinese was granted by the authors of the English EBP^2^Q. In accordance with established guidelines [33], the following steps were implemented. Firstly, two bilingual postgraduate nursing students independently forward-translated the EBP^2^Q from English to Chinese. Secondly, a synthesized version was completed after careful discussion with a third bilingual postgraduate nursing student. Thirdly, the synthesized version was independently back-translated by two other bilingual translators with no knowledge of the original EBP^2^Q. These translators were native Chinese speakers, each with a year study experience in Britain to achieve a Master’s degree in Nursing at the University of Salford, Manchester, United Kingdom. Fourthly, the two overseas translators who had studied in Britain and a bilingual nursing expert reviewed and compared the two back-translation versions and agreed on an integrated back-translation version. The research team (including professionals in the fields of nursing, statistics, and questionnaire development) subsequently verified and resolved discrepancies amongst the forward and backward translations, as well as implemented feedback from the original authors to produce a pre-final Chinese version. This version was considered the semantic and conceptual equivalent of the English EBP^2^Q.

##### Expert committee review and content validation

A panel of six experts was formed to determine the need for adaptations and item removal to ensure compatibility in Chinese populations. The experts were educated to at least Master’s degree level and included clinical nursing experts, EBP experts, and nursing research educators with senior academic titles. In addition to academic qualifications, the experts grew up, were educated, and have worked in hospitals in different provinces, potentially representative of the health, social, and cultural systems of Mainland China (see Additional file 1). The panel evaluated the relevance, clarity, and the cultural and semantic equivalence of each item on three separate four-point Likert scales (relevance: 1-not relevant, 4-highly relevant; clarity: 1-very unclear- needs total revision, 4-highly clear-does not need revision; equivalence: 1-completely different, 4-completely equivalent) and made modification suggestions [34].

##### Pilot testing

Thirty registered nurses, employed at Nanfang Hospital, Southern Medical University (Guangzhou, China), volunteered to participate in the pilot test of the translated 58-item Chinese version of the EBP^2^Q, which was conducted in line with the recommended sample size of 10–40 participants [35]. The questionnaire completion time was recorded and participants were interviewed on their understanding of each item, and asked for advice on the wording of the items and on further ways to promote the usability of the pilot instrument.

#### Data analysis

Descriptive statistics were used to calculate the mean time required by participants to complete the questionnaire. We used the content validity index (CVI) to evaluate the validity of the quantified content in the scale. A scale-level CVI (S-CVI) > 0.90, and an item-level CVI (I-CVI) > 0.83 were considered appropriate [34].

### Methods in stage 2: testing of the psychometric properties of the EBP^2^Q

#### Procedure

##### Participants

A non-probabilistic sampling of convenience was used. Prior to recruitment, authorization was obtained from the head nurses in each of the clinical departments. Eligible participants were registered nurses employed at Nanfang Hospital, Southern Medical University. The nurses were informed verbally by the head nurses regarding the survey in advance during the shifts of nurses or at a departmental meeting. The names of the nurses who were willing to participate in the research were collected by the head nurses and forwarded to the research team. Using scripted instructions (including the unified greeting, self-introduction and survey procedure), the research team subsequently contacted the volunteers before or after the changes in shift in a department meeting room or lounge. The nurses were provided with an information sheet explaining the purpose of the survey and instructions on how to complete the questionnaire. The volunteer nurses were assured that the survey was anonymous and confidential, and that they could withdraw at any time without consequences. All nurses who agreed to participate signed an informed consent form. Subsequently, the research team distributed the questionnaires to the participating nurses in a department meeting room or lounge. The questionnaires took approximately 20 min to complete. Once completed, the questionnaire was handed to the investigators who were waiting outside. If the participating nurses could not complete the questionnaire immediately, they could also complete it at home, and return it to the head nurses within the following 3 days. The research team collected the remaining questionnaires 10 days later.

##### Construct validity-structural validity

A cross-sectional validation study using two independent convenience samples of subjects with a total of 580 clinical nurses was conducted in two periods (exploratory factor analysis [EFA] and confirmatory factor analysis [CFA]) between September 2018 and April 2019. The CFA was necessary due to the different factor structure from the original English EBP^2^Q, as identified in the EFA. The recommended sample size to perform the EFA is 5–10 fold larger than the number of items in the questionnaire [36]. Therefore, a sample of 290 participants was required for testing, with 330 participants recruited between September and November 2018. For the CFA, 250 participants (exceeding the minimum acceptable number of 200 in the CFA) [37], were recruited between February and April 2019.

Prior to the EFA, the same 330 clinical nurses were tested for item analysis to identify each of the items, and those failing the standards were meant to be removed.

##### Construct validity-convergent validity

A convenience sample of 122 participants also completed the Chinese EBPQ [31] at the second period (CFA) to assess the convergent validity of the revised Chinese EBP^2^Q.

##### Criterion validity

The Chinese EBP^2^Q was tested for the ability to separate groups by subscale scores based on the highest level of education, present position, EBP training, experience in conducting research, and level of English.

##### Test-retest reliability

Twenty-two nurses who completed the Chinese EBP^2^Q for the CFA voluntarily completed the questionnaire again 2 weeks later to evaluate the test-retest reliability.

#### Data analysis

##### Item analysis of the first and revised second versions of the Chinese EBP^2^Q

The item analysis of the Chinese EBP^2^Q for the EFA and the revised Chinese EBP^2^Q after the CFA was calculated to test the following three criteria: (1) item discriminability, the Critical ratio (CR) index was expected to reach a value of > 3.0 [38]; (2) item homogeneity, item-total correlation was expected to be > 0.3 [39]; (3) Cronbach’s α after deleting the item was expected to be smaller than the corresponding subscale [40].

##### Construct validity-structural validity

The EFA and CFA were applied to establish the structural validity of the Chinese version of the EBP^2^Q. As recommended by Hair et al. [41], principle component analysis with varimax rotation was employed in the EFA to determine the structure of the questionnaire. Factors extracted and items reserved complied with the following rules: (1) eigenvalue > 1; (2) factor loading > 0.50; (3) no cross-loading items with factor loading ≥0.40 [41]; (4) ≥3 items belonging to each factor [42]. The CFA with maximum likelihood estimation was performed following the EFA. Several model fit indices were checked: (1) the result of the Chi-squared test divided by the degrees of freedom *(χ*^*2*^*/df*) was expected to be smaller than 3.0; (2) the standardized root mean square residual (SRMR) was expected to be smaller than 0.08; (3) the comparative fit index (CFI) was expected to be larger than 0.90 [43]; and (4) the root mean squared error of approximation (RMSEA) values < 0.01, < 0.05 and < 0.08 indicate excellent, good, and mediocre fit, respectively [44].

##### Construct validity-convergent validity

Sixteen comparable items of the Chinese version of the EBPQ [31], which involved three domains (i.e., *Sympathy*, *Confidence*, and *Practice)* were matched to the corresponding items of the revised Chinese EBP^2^Q after the CFA to evaluate convergent validity. Considering the non-normality of the data, the Spearman’s correlation coefficient was calculated to compare the participants’ scores on the two questionnaires, on both domains and items.

##### Criterion validity

To assess criterion validity, t-tests respectively one-way analyses of variance regarding the five criteria described above were performed based on the mean scores of each subgroup on the domains. Post-hoc analyses were conducted using the Bonferroni-adjusted significance test controlling for Type І error to identify differences between sample means.

##### Internal consistency

Analysis of internal consistency was applied to the entire scale and the domains of the revised Chinese EBP^2^Q after the CFA. Cronbach’s α ≥0.70 was deemed acceptable [45]. The composite reliability estimated in the CFA was also expected to be ≥0.70 [46].

##### Test-retest reliability

For test-retest reliability, the ICCs of the items and the domains of the revised Chinese EBP^2^Q after the CFA were calculated within 2-week intervals. ICC values ≥0.75 and 0.40–0.74 denoted perfect and adequate reliability, respectively [47].

Descriptive statistics were calculated for participant characteristics, items and scales. The ceiling and floor effect of the revised Chinese EBP^2^Q after the CFA were tested in terms of the lowest and highest item means and standard deviation (SD).

All statistical analyses were performed using IBM SPSS version 20.0 and IBM AMOS version 22.0. *P-values* < 0.05 were considered statistical significance.

## Results

### Results of stage 1: translation and adaptation of the EBP^2^Q

#### Translation

In the forward-backward translation process from the original EBP^2^Q, most of the changes were related to terms and phrases that were meaningful in the context of Chinese culture. For example, in item 6, the term ‘develop’ was replaced by ‘learn’; the terms ‘accessing’, ‘acquiring’, and ‘appraising’ were replaced by ‘searching’, ‘retrieving’ and ‘evaluating’, respectively; and the term ‘area’ was replaced by ‘field’. In item 18, the phrase ‘In making decisions about my professional work … ’ was replaced by ‘When making professional decisions … ’. Other changes from the original to the Chinese version were ‘Workplace experience … ’ to ‘Experience from work practices or colleagues … ’(original item 19), ‘real world’ to ‘actual work’ (original item 21), ‘in your workplace’ to ‘at work’ (original item 46), ‘research reports’ to ‘research literature’ (item 45), and where used throughout the questionnaire, ‘client’ was changed to ‘patient’. There were also changes to some labels on the Likert scale: ‘true’ became ‘correct’; ‘neutral’ became ‘not so sure’; and ‘one month’, ‘once a fortnight’ and ‘once a week’ became ‘seldom’, ‘sometimes’, and ‘frequently’, respectively.

#### Content validation by the expert committee

The expert scores for content validity on the S-CVI for relevance, clarity, and equivalence were 0.99, 0.98, and 0.98, respectively. The I-CVI of all the items for relevance, clarity, and equivalence ranged between 0.83 and 1.00 (see Additional file 2).

#### Pilot testing

All participants completed the pre-test instrument in a mean time of 12.27 min (SD: 2.46 min; range: 8–19 min). Based on feedback, the acronym “EBP” was replaced with the full term “Evidence-based practice” to ensure the easy understanding and appropriateness of the questionnaire in practice.

### Results of stage 2: testing of the psychometric properties of the EBP^2^Q

#### Participants

In the first period of the EFA, 303 of the 330 questionnaires distributed were included in the data analysis. Of the 27 excluded questionnaires, nine were missing and 18 were incomplete (> 25% of items not completed). This represented a response rate of 91.8%. For the CFA, 240 of the 250 questionnaires distributed were included (three questionnaires were missing and seven were incomplete), representing a response rate of 96.0%. “Hot Deck” imputation was used for missing data in questionnaires with ≥75% of items completed [48].

Table 1 presents the sociodemographic and EBP-related characteristics of the total sample of 543 nurses. The mean age of the nurses was 30.8 years (range: 22–60 years), and 91.0% were females. More than two-thirds of the nurses (87.5%) held a Bachelor’s degree as their highest education qualification. Half of the nurses (52.1%) practiced in the surgery department. The vast majority of the nurses (71.5%) had junior professional titles. Approximately one-third (35.7%) had been working for > 5–10 years. The majority were staff nurses (91.2%), while the remaining (8.8%) were nurse administrators. Although nearly half of the nurses did not have EBP training experience (44.8%), 10.1% of nurses had undertaken > 20 h of EBP training. Also, 69.8% of the nurses had some basic knowledge of English. The majority had never conducted a research study (91.0%).

**Table 1:** Demographic Characteristics of nursing staff (*n* = 543)

	Characteristics	Number (%)
Age	years (mean, SD)	30.76 (5.53)
Sex	Female	494 (91.0)
	Male	49 (9.0)
Department	Internal Medicine	214 (39.4)
	Surgery	283 (52.1)
	Gynecology & Pediatrics	30 (5.5)
	Others	16 (2.9)
Technical Title	Junior	388 (71.5)
	Intermediate	137 (25.2)
	Senior	18 (3.3)
Working Time	≤5 years	187 (34.4)
	> 5–10 years	194 (35.7)
	> 10 years	162 (29.9)
Education	Diploma	34 (6.3)
	Bachelor	475 (87.5)
	Master	34 (6.3)
Position	Nurse administrator	48 (8.8)
	Staff nurse	495 (91.2)
Received EBP Training	No	243 (44.8)
	≤20 h	245 (45.1)
	> 20 h	55 (10.1)
English Level	None	164 (30.2)
	ETT/CET-4	308 (56.7)
	CET-6/IELTS/TOEFL	71 (13.1)
Conducted a Research Study	No	494 (91.0)
	Yes	49 (9.0)

Note: ETT, English for Technical Title; CET-4, College English Test-Band 4; CET-6, College English Test-Band 6; IELTS, International English Language Test System; TOEFL, Test of English as A Foreign Language; EBP, Evidence-Based Practice

#### Item analysis of the first version of the Chinese EBP^2^Q for the EFA

The CR values of the items were all significant (*p* < 0.01). Only item 15 (EBP does not take into account the limitations of my daily work) showed a CR value of < 3.0. The item-total correlations were all > 0.30, with the exception of item 15 (EBP does not take into account the limitations of my daily work: *r* = 0.17) and item 19 (Experience from work practices or colleagues is the most reliable and effective way to solve problems: *r* = 0.26). Except for items 19 (Experience from work practices or colleagues is the most reliable and effective way to solve problems), 49 (Computer skills), and 50 (Ability to identify your knowledge gaps), when there was separate deletion of all other items, the α did not increase to be larger than the α of the corresponding subscale. Based on this analysis, items 15, 19, 49, and 50 were removed, leaving 54 items for evaluation in the EFA (see Additional file 3).

#### Structural validity-EFA

The remaining 54 items were analyzed using the EFA. Prior to the EFA, two factor analyses criteria were assessed: the Kaiser–Meyer–Olkin measure was 0.932 and the Barlett’s test of sphericity was significant (*p* < 0.001), which indicated fitness for the EFA [49]. The first EFA attempt extracted 10 factors, explaining 72.70% of the total variance. However, based on the item retention rule that items should not be cross-loaded on two different factors with loading ≥0.4, the following eight items were removed: 24 (systematic review), 47 (formally share and discuss literature/research findings with others in your department/practice, e.g. journal clubs, achievement report speech/experience sharing meeting), 25 (odds ratio), 36 (dichotomous outcomes), 34 (clinical importance), 35 (randomized controlled trial), 32 (statistical significance), and 30 (forest plot) were identified to be removed. Following this, a re-run of the model showed that two items (22, relative risk and 23, absolute risk) formed naturally into an individual factor, violating the rule of three items per factor. Subsequently, item 23 (absolute risk), with a relatively high factor loading, was removed first. After re-analysis, an eight-factor solution was identified, with an explained variance of 71.0% from a total of 45 items (see Additional file 4). Moreover, the EFA of all the 45 items reached factor loadings > 0.50, ranging between 0.55 and 0.83. The domains were renamed (maintaining initial item numbers) according to their common characteristics to summarize the concepts of items in each of the eight domains in the revised structure, e.g. *Basic Understanding* describes an individual’s fundamental conception of EBP, *Intention* refers to the individual’s determination to strengthen EBP competencies, *Attitude* means an individual’s emphasis or values about EBP in practical work ......(See Table 2).

**Table 2:** Structure of the 45-item Chinese Evidence-Based Practice Profile Questionnaire^a^

Domain	Item Numbers^b^	Description
Basic understanding	1, 2, 3,4	Fundamental conception of EBP
Intention	5, 6, 7, 8	Determination to strengthen EBP competencies
Attitude	9, 10, 11, 12, 13, 14	Emphasis or values about EBP in practical work
Sympathy	16, 17, 18, 20, 21	Compatibility between EBP and practicality or feasibility in day-to-day work or occupation
Clinical-related terms	22, 27, 31, 33, 37, 38	Terminology used most in clinical practice research
EBP related terms	26, 28, 29	Terminology related to the EBP study
Practice	39, 40, 41, 42, 43, 44, 45, 46	Application of EBP in clinical circumstance
Confidence	48, 51, 52, 53, 54, 55, 56, 57, 58	Individual’s perception of their EBP skills

^a^All items were rated on a five-point Likert scale; ^b^Item numbers relate to the original Evidence-Based Practice Profile Questionnaire

*EBP* Evidence-Based Practice

#### Structural validity-CFA

**Fig. 1:**
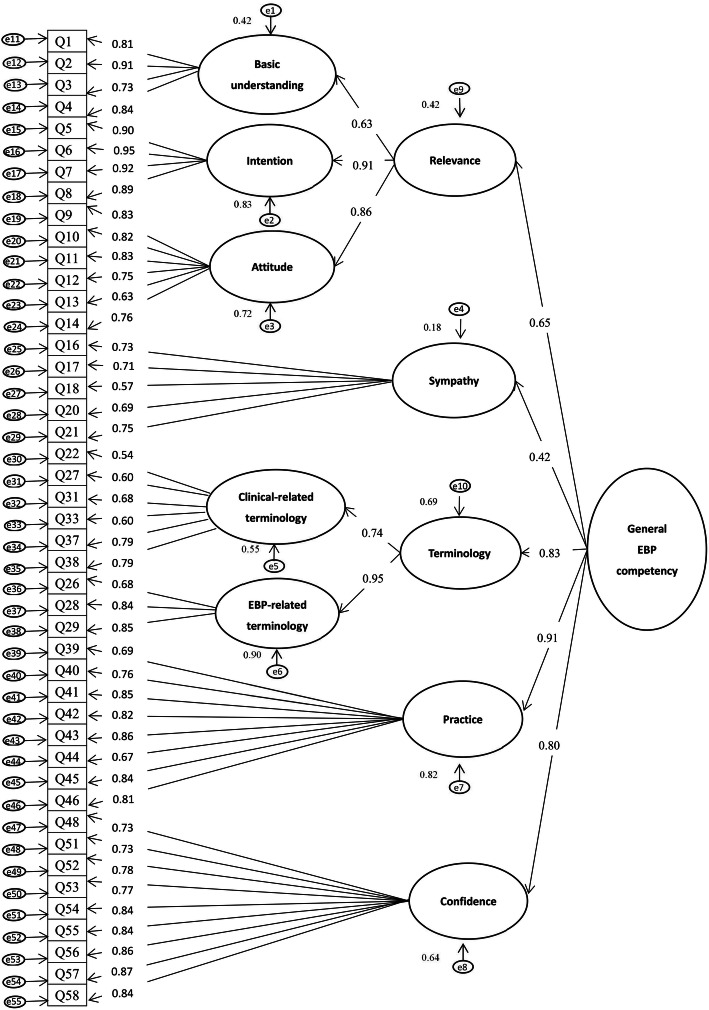
Confirmatory factor analysis of the 45-item Chinese Evidence-Based Practice Profile questionnaire (*n* = 240)

Considering the five-factor structure in the original EBP^2^Q, we tried a three-order factor model test in the CFA to verify the prior EFA solution. The results revealed acceptable goodness-of-fit indices: *χ*^*2*^*/df* = 2.001; RMSEA = 0.065; SRMR = 0.077; and CFI = 0.884. With one exception, the estimated parameters of all the items and factors were statistically significant (*p* < 0.001), with all standardized factor loadings > 0.50 (ranging between 0.54 and 0.95): *Basic Understanding*, 0.73–0.91; *Intention,* 0.89–0.95; *Attitude,* 0.63–0.83; *Sympathy,* 0.57–0.75; *EBP-related Terminology,* 0.68–0.85; *Clinical-Related Terminology,* 0.54–0.79; *Practice,* 0.67–0.86; *Confidence,* 0.73–0.87; *Relevance,* 0.65–0.91; *Terminology,* 0.74–0.95; *General EBP Competency,* 0.42–0.91. The one exception was the loading of the first-order factor *Sympathy* to the third-order factor *General EBP Competency* (0.42, *p* < 0.001) (Fig. 1).

#### Convergent validity

For convergent validity, the correlation coefficients between the comparable individual items in the two questionnaires ranged between 0.19 and 0.52 for *Practice*, 0.27 and 0.27 for *Sympathy*, and 0.42 and 0.65 for *Confidence*. While statistically significant for all, the correlation of each of the three comparable summed factor scores was moderate for *Practice* (*r =* 0.58, *p* < 0.001) and *Confidence* (*r =* 0.68, *p* < 0.001) and low for *Sympathy* (*r =* 0.32, *p* = 0.001).

#### Criterion validity

All mean factor scores of the nurses on the eight domains were significantly different in the subgroups regarding the educational background, EBP training, research study experience, and level of English (*p* < 0.05). In terms of the nurses’ present position, the mean factor scores of the nurse administrators were significantly different from those of the staff nurses in the *Basic understanding*, *Intention*, *Attitude*, *EBP-related terms*, and *Practice* domains (*p* < 0.05). In the post-hoc analysis, nurses who had undertaken > 20 h EBP training and had a Master’s degree and College English Test-Band 6/International English Language Test System/Test of English as A Foreign Language (CET-6/IELTS/TOEFL) qualifications scored significantly higher than all the other nurses in each of the eight domains (*p* < 0.05) (Table 3).

**Table 3:** Differences in the Revised Chinese Evidence-Based Practice Profile Questionnaire by nurses characteristics (n = 543)

Characteristics	Number	Basic understanding	Intention	Attitude	Sympathy	Clinical related terms	EBP related terms	Practice	Confidence
**Education**									
*Diploma (A)*	34	2.71 ± 0.84	3.30 ± 0.60	3.70 ± 0.52	2.89 ± 0.54	2.51 ± 0.78	1.96 ± 0.83	2.30 ± 0.73	2.10 ± 0.67
*Bachelor (B)*	475	2.92 ± 0.83	3.55 ± 0.87	3.93 ± 0.60	3.19 ± 0.68	2.50 ± 0.76	2.20 ± 0.91	2.39 ± 0.80	2.04 ± 0.73
*Master (C)*	34	3.74 ± 0.70	4.42 ± 0.59	4.32 ± 0.68	3.56 ± 0.81	3.12 ± 0.82	3.71 ± 0.91	3.39 ± 0.70	2.99 ± 0.79
F		17.17***	36.906***	9.659***	8.570***	10.394***	45.685***	26.243***	27.126***
Post-hoc analysis		*C > B, C > A*	*C > B > A*	*C > B > A*	*C > B > A*	*C > B, C > A*	*C > B, C > A*	*C > B, C > A*	*C > B, C > A*
**Received EBP Training**									
*Non (A)*	243	2.55 ± 0.88	3.24 ± 0.90	3.73 ± 0.62	3.06 ± 0.69	2.26 ± 0.73	1.81 ± 0.75	2.03 ± 0.69	1.8 ± 0.62
*≤ 20 h (B)*	245	3.20 ± 0.60	3.77 ± 0.73	4.06 ± 0.55	3.22 ± 0.64	2.71 ± 0.72	2.51 ± 0.91	2.69 ± 0.72	2.26 ± 0.71
*> 20 h (C)*	55	3.69 ± 0.75	4.36 ± 0.55	4.38 ± 0.50	3.67 ± 0.69	3.03 ± 0.80	3.36 ± 0.97	3.2 ± 0.82	2.75 ± 0.73
F		67.794***	72.292***	38.059***	18.830***	36.464***	87.050***	86.380***	45.634***
Post-hoc analysis		*C > B > A*	*C > B > A*	*C > B > A*	*C > B > A*	*C > B > A*	*C > B > A*	*C > B > A*	*C > B > A*
**English Level**									
*Non (A)*	164	2.74 ± 0.87	3.23 ± 0.92	3.77 ± 0.61	3.06 ± 0.62	2.47 ± 0.78	1.83 ± 0.77	2.19 ± 0.71	1.91 ± 0.66
*ETT/CET-4 (B)*	308	3.00 ± 0.82	3.67 ± 0.80	3.98 ± 0.60	3.19 ± 0.71	2.52 ± 0.76	2.30 ± 0.92	2.45 ± 0.81	2.13 ± 0.76
*CET-6/IELTS/TOEFL (C)*	71	3.30 ± 0.83	4.10 ± 0.72	4.19 ± 0.56	3.51 ± 0.67	2.80 ± 0.80	3.23 ± 0.98	3.04 ± 0.82	2.46 ± 0.85
F		11.656***	29.751***	13.906***	11.008***	4.564*	60.314***	29.003***	12.757***
Post-hoc analysis		*C > B > A*	*C > B > A*	*C > B > A*	*C > B, C > A*	*C > B, C > A*	*C > B > A*	*C > B > A*	*C > B > A*
**Conducted a Research Study**									
*no*	494	2.88 ± 0.83	3.52 ± 0.86	3.90 ± 0.60	3.17 ± 0.68	2.50 ± 0.76	2.16 ± 0.91	2.36 ± 0.78	2.03 ± 0.71
*yes*	49	3.73 ± 0.66	4.28 ± 0.68	4.35 ± 0.58	3.46 ± 0.69	3.0 ± 0.81	3.47 ± 0.86	3.37 ± 0.69	2.86 ± 0.80
t		−7.009***	−5.937***	−5.020***	−2.885**	−4.411***	−9.610***	−8.768***	− 7.662***
**Position**									
*Staff nurse*	495	2.92 ± 0.85	3.55 ± 0.87	3.91 ± 0.62	3.18 ± 0.68	2.53 ± 0.79	2.23 ± 0.97	2.40 ± 0.82	2.09 ± 0.77
*Nurse administrator*	48	3.31 ± 0.80	4.06 ± 0.75	4.24 ± 0.47	3.28 ± 0.73	2.64 ± 0.65	2.84 ± 0.94	2.90 ± 0.71	2.29 ± 0.66
t		−3.054**	− 3.972***	− 3.584***	−0.91	−1.004	− 4.199***	− 4.038***	− 1.763

Notes: ETT, English for Technical Title; CET-4, College English Test-Band 4; CET-6, College English Test-Band 6; IELTS, International English Language Test System; TOEFL, Test of English as A Foreign Language; EBP, Evidence-Based Practice;& If one has conducted a research study

***p < 0.001; **p < 0.01; *p < 0.05

#### Internal consistency and test-retest reliability

The overall Cronbach’s α for the scale was 0.96. As listed in Table 4, the α for the internal consistency of the eight domains ranged between 0.85 and 0.95, with the composite reliability from the CFA ranging between 0.82 and 0.95, indicating good internal consistency. The ICCs for the items ranged between 0.50 and 0.91 and for the domain between 0.75 and 0.96, revealing sufficient time stability.

**Table 4:** Internal consistency and Test-retest reliability of the Revised Chinese Evidence-Based Practice Profile Questionnaire

Domain	Mean (SD)	Cronbach’s α	Composite Reliability	Range Item ICC	Domain ICC
Basic understanding	3.02 (0.86)	0.91	0.89	0.73–0.86	0.84
Intention	3.68 (0.84)	0.95	0.95	0.67–0.83	0.80
Attitude	4.02 (0.60)	0.90	0.90	0.67–0.80	0.91
Sympathy	3.23 (0.71)	0.85	0.82	0.50–0.69	0.75
Clinical related terms	2.53 (0.78)	0.85	0.83	0.68–0.87	0.90
EBP related terms	2.36 (0.99)	0.87	0.84	0.80–0.91	0.90
Practice	2.52 (0.81)	0.92	0.93	0.57–0.84	0.84
Confidence	2.17 (0.77)	0.95	0.94	0.75–0.90	0.96

Note: EBP, Evidence -Based Practice; ICC, Intraclass correlation coefficient

#### Item analysis and the ceiling-floor effect of the revised 45-item Chinese EBP^2^Q

Regarding the item analysis of the revised 45 items, with the exception of items 26 (meta-analysis) and 44 (consider patients’ preferences when making clinical/professional decisions), where α if the items were deleted was not lower than the α for the corresponding domains, the rest all met the requirement. All results from the item-total correlation analysis were significant (*p* < 0.01), and the coefficients ranged between 0.32 and 0.74. Descriptive statistic results showed that the lowest and highest item means were 1.86 (SD: 0.91) and 4.13 (SD: 0.72), respectively, indicating the absence of ceiling-floor effect (see Additional file 5).

## Discussion

The EBP^2^Q was initially designed to evaluate EBP profile across a range of professions and different levels of experience in Australian populations, and has demonstrated strong psychometric properties [28]. Guided by the established guidelines [33], the EBP^2^Q was translated and cross-culturally adapted into Chinese and validated using a sample of 543 clinical nurses. Our psychometric tests highlighted the capability of the EBP^2^Q-C to assess the EBP knowledge, attitude, skills, and behavior in domestic nursing practice, providing evidence of valid measurement properties of the instrument.

Concerning content validity, the S-CVI and I-CVI for each of the individual items reached acceptable values, with all S-CVI values > 0.90 for relevance, clarity, and equivalence, and all I-CVI values ≥0.83. These findings suggest that the items conform well to the conceptual framework.

Based on the first item analysis, four items were deleted because they did not fulfil the threshold standards, leaving 54 items subsequently analyzed in the EFA. The EFA resulted in the removal of nine items based on a priori item retention rules. This led to a change in the questionnaire from a five- to an eight-factor structure. The differences in the two structures may have resulted from the late introduction of an EBP culture in nursing in China and the less well-developed understanding of EBP by clinical nurses [5]. The three-order factor model in the CFA conducted to verify the reformed structure of the revised 45 items demonstrated a comparably good model of fit. An exception was the CFI (0.884), which was slightly lower than the recommended criterion of 0.90, but still approached the value for an acceptable fit. As for the lower factor loading of “*Sympathy*” to “*General EBP Competency*” (0.42), the possible reason may be that under the complex situation in China, the daily workload of clinical nurses is so heavy that they don’t have enough time to carry out the EBP projects, so that the “*Sympathy*” can not effectively reflect their “*General EBP Competency*”. In general, based on the EFA and CFA, items were distributed accordingly to, and positively correlated with, the corresponding domains.

The 58 domain items from the original EBP^2^Q were reduced by 13 items to 45 items for the EBP^2^Q-C. In the item analysis stage, four items were removed based on the analysis of the tested sample showing that items may be measuring a different concept or could be misleading. A further nine items were removed in the EFA stage because they did not adequately correlate with the eight subscales identified. Nevertheless, the reduction of the items enables a more economic measurement. Eight of the 17 items in the domain of *Terminology* in the original EBP^2^Q were removed. It may be that these eight terminology items are not meaningful to the Chinese population. Nine of the original 11 items in the EBP^2^Q *Confidence* domain were included in the EBP^2^Q-C with the exclusion of ‘computer skills’ and ‘ability to identify gaps in knowledge’ (item 50). It may be that item 50 is not considered separate from item 51 where the identified gap or information need is transferred into a clearly answerable question. Importantly, the formulation of a clearly answerable question, which is an important concept in EBP, remains in the new version. Only one item (formal sharing of research findings) from nine of the *Practice* domain in the original EBP^2^Q was not included in the same domain of the Chinese version. The included items appear to adequately cover the application of EBP in clinical circumstances. The seven items for the domain of *Sympathy* in the original EBP^2^Q were reduced to five items in the EBP^2^Q-C. The two removed original items were item 15 (regarding whether EBP takes into account the limitations of daily work), and item 19 (rating the role of experience from work practice and colleagues in decision-making). It is possible that in the Chinese context, the subtle differences between items 19 and 18 (which rate the value of clinical experience in professional decisions compared with research experience) are not recognized. Overall, it appears that the domains in the new questionnaire adequately cover those under investigation. However, caution should be exercised in comparing study results where the two different questionnaires (Chinese and original) are used, due to changes based on differences in Chinese culture, workplace relationships, and linguistic nuances.

The findings for the convergent validity of the EBP^2^Q-C with the Chinese EBPQ as criterion (*Practice:* 0.58; *Sympathy:* 0.32; *Confidence:* 0.68) suggested adequate convergent validity except for *Sympathy* in contrast to the convergent validity of the original EBP^2^Q with the EBPQ as criterion (*Practice:* 0.66; *Sympathy:* 0.54; *Confidence:* 0.80) [28]. The questionnaire revisions for the EBP^2^Q-C in terms of language translation, cultural interpretation, and item reduction, as discussed above for all domains and specifically in each of the three comparative domains with the EBPQ, may have contributed to these weaker correlations.

Regarding the criterion validity, all the comparisons between five key characteristics showed statistically significant differences. Nurses with higher education, more extensive EBP training, experience in conducting research study, and better level of English scored significantly higher on each of the eight individual domains. These findings were consistent with the verified associations of these sociodemographic variables with EBP domains reported in previous studies [9, 50, 51]. However, compared with the statistical significance of EBP training on all the eight domains in the EBP^2^Q-C, the results in the Polish [29] and Norwegian [30] version only demonstrated significance in the *Relevance, Terminology,* and *Confidence* or *Sympathy* domains*.* This may be due to differences in the various EBP training times, with options limited to yes/no only in the Norwegian version, and none and 12 h in the Polish version. In the current study, there were three levels with greater differences in exposure (none, ≤20 h, and > 20 h). This may indicate that the duration of exposure to EBP training is of great importance to the effectiveness of the training program at a self-reporting level. As presented in Table 3, nurses in the role of administrators had significantly higher mean values in five of the domains, with significance not reached for the domains of *Sympathy, Clinical-related terms*, and *Confidence*. Previous research has also demonstrated that nurses who hold higher-level positions reported better values in the EBP domains [50]. Results from the post-hoc analysis also confirmed the significant influence of the Master’s degree, EBP training for > 20 h, and CET-6/IELTS/TOEFL qualifications on the EBP competencies of nurses. The results of the present study showed significant differences of these key characteristics in different EBP domains. Hence, these data demonstrated the validity of the EBP^2^Q-C to assess the self-reported EBP in Chinese clinical nurses with different training.

For internal consistency, the composite reliability from the CFA was employed as an additional supplementary analysis. It has been previously reported that the Cronbach’s α coefficient can underestimate or overestimate reliability [52]. For the newly proposed domains in the EFA, both the observed estimators exceeded the recommended standard, similar to that noted in the English EBP^2^Q [28], supporting the internal consistency. The temporal stability of the EBP^2^Q-C was confirmed by the test-retest reliability with a 2-week interval separating the completion of the two questionnaires. The ICC for the items exceeded 0.40, and reached satisfactory values (≥0.75) for all domains, with similar values to those obtained from the English EBP^2^Q [28].

In the item analysis of the 45-item EBP^2^Q-C (see Additional file 5), all item-to-total correlations were statistically significant and indicated a stronger association with the total scale. These findings demonstrate the homogeneity of the revised 45-item Chinese questionnaire. Although the results of changes in α when items are sequentially excluded from the analysis recommended the removal of items 26 (meta-analysis) and 44 (consider patients’ preferences when making clinical/professional decisions), these two items were retained to ensure the comprehensiveness of the instrument. As stated in the definition of EBP [14], health care professionals should instinctively take the patients’ values, expectations, and preferences into consideration to ensure evidence-based health care. Therefore, reserving item 44 (consider patients’ preferences when making clinical/professional decisions) may assist in assessing the EBP behavior of participants.

Descriptive statistics of the EBP^2^Q-C showed that nurses scored highest in the *Attitude* domain and lowest in the *Confidence* domain. The findings suggested that, while nurses held a positive attitude towards the EBP, they still lacked the necessary EBP competencies and confidence to incorporate research evidence into professional practice. This was similar to findings reported in recent studies involving nurses in Turkey and the USA [11, 53]. These findings suggest that there is ‘a long way to go’ for domestic EBP educators and training mentors in tailoring efficient instructional modes for clinical nurses. The floor and ceiling effects of each item in the EBP^2^Q-C were explored using the lowest and highest item mean (1.86 [SD: 0.91] and 4.13 [SD: 0.72], respectively), demonstrating the absence of the ceiling or floor effect (see Additional file 5). McEvoy et al. reported similar results for the EBP^2^Q: 1.71 (SD: 1.0) and 4.09 (SD: 0.9), respectively [28].

### Limitations and considerations for further research

Despite the satisfactory findings, the current study was characterized by a number of limitations. Firstly, while the sample size was sufficient for the measurement of the tested properties, all nurses were recruited from a single tertiary hospital through convenience sampling: this may restrict the broader application of the study findings. Further research should involve hospitals representing different levels using the stratified random sample method, to expand the generalizability of the results. Secondly, all data were self-reported, which may result in the overestimation or underestimation of actual competence of the respondents, thus leading to reporting bias. Thirdly, the responsiveness of the EBP^2^Q-C in EBP educational or training programs is unknown. This may be valuable to consider in future validation research.

Finally, the EBP^2^Q-C was validated only with nurses in contrast to the original EBP^2^Q where a range of professions were included in the development and validity-testing. Further research may start with other professionals such as clinical doctors.

## Conclusion

This study provides preliminary evidence for the EBP^2^Q-C as a psychometrically robust tool for the evaluation of EBP in nurses in China. Although consistent in terms of conceptualization, the factor structure of the EBP^2^Q-C differed from that of the English version, which necessitated further validation of the instrument. The final revised rigorously developed 45-item EBP^2^Q-C has an eight-factor structure and demonstrated acceptable structural, convergent and criterion validity, test-retest reliability, and internal consistency. The EBP^2^Q-C may be used in EBP education or training programs to improve the skills of participants, either as self-assessment or an outcome measurement of learning. It may also be used in the design of EBP courses by clinical EBP educators, to develop efficient evaluations of education or training programs.

## Supplementary information

**Additional file 1.** Characteristics of the Experts in Content Validity of the Questionnaire (n = 6).

**Additional file 2.** Item level content validity index (I-CVI) of the pre-final version of EBP^2^Q (n = 6).

**Additional file 3.** Item Analysis of the 58-item Evidence-Based Practice Profile Questionnaire (n = 303).

**Additional file 4.** Exploratory factor analysis of the 54-item Chinese Evidence-Based Practice Profile Questionnaire (n = 303).

**Additional file 5.** Item Analysis of the Revised 45-item Chinese Evidence-Based Practice Profile Questionnaire (n = 303).

## Data Availability

The datasets used and/or analyzed during the current study are available from the corresponding author on reasonable request.

## Abbreviations

EBP: Evidence-Based Practice
EBP^2^Q: Evidence-Based Practice profile questionnaire
EBP^2^Q-C: the Chinese version of Evidence-Based Practice profile questionnaire
EFA: Exploratory factor analysis
CFA: Confirmatory factor analysis
EBPQ: Evidence-Based Practice questionnaire
I-CVI: Item level content validity index
S-CVI: Scale level content validity index
ICC: Intraclass correlation coefficient
CR: Critical ratio
RMSEA: Root mean square error of approximation
SRMR: Standardized root mean square residual
CFI: Comparative fit index
KMO: Kaiser-Meyer-Olkin
EBN: Evidence-Based Nursing
JBI: Joanna Briggs Institute
MSN: Master of Science in Nursing
CNA: Chinese Nursing Association

## References

[1] DiCenso A, Guyatt G, Ciliska D (2014). Evidence-based nursing-E-book: a guide to clinical practice.

[2] Skaggs M, Daniels JF, Hodge AJ, DeCamp VL (2018). Using the evidence-based practice service nursing bundle to increase patient satisfaction. J Emerg Nurs.

[3] Baker JD (2017). Nursing research, quality improvement, and evidence-based practice: the key to perioperative nursing practice. AORN J.

[4] Saunders H, Vehvilainen-Julkunen K (2016). The state of readiness for evidence-based practice among nurses: an integrative review. Int J Nurs Stud.

[5] Zhao J, Liu X, Zhang W, Xing Y, Cho SW, Hao Y (2018). Evidence-based nursing outputs and hot spot analysis of the last 5 years in mainland China: results of a bibliometric analysis. Int J Nurs Pract.

[6] Farokhzadian J, Khajouei R, Ahmadian L (2015). Evaluating factors associated with implementing evidence-based practice in nursing. J Eval Clin Pract.

[7] Tolera BD, Feng H (2017). Assessment of attitudes, skills and source of knowledge on utilization of EBP among registered nurses in Xiangya Hospital of Central South University, Changsha, China. Am J Nursing Sci.

[8] Melnyk BM, Gallagher-Ford L, Long LE, Fineout-Overholt E (2014). The establishment of evidence-based practice competencies for practicing registered nurses and advanced practice nurses in real-world clinical settings: proficiencies to improve healthcare quality, reliability, patient outcomes, and costs. Worldviews Evid-Based Nurs.

[9] Tomotaki A, Fukahori H, Sakai I (2020). Exploring sociodemographic factors related to practice, attitude, knowledge, and skills concerning evidence-based practice in clinical nursing. Jpn J Nurs Sci.

[10] Lam CK, Schubert C (2019). Evidence-based practice competence in nursing students: an exploratory study with important implications for educators. Worldviews Evid-Based Nurs.

[11] Kilicli AB, Kelber ST, Akyar I, Litwack K (2019). Attitude, source of knowledge, and supporting factors on evidence-based nursing among cardiovascular nurses: a cross-sectional descriptive study in Turkey. J Eval Clin Pract.

[12] Wu Y, Brettle A, Zhou C, Ou J, Wang Y, Wang S (2018). Do educational interventions aimed at nurses to support the implementation of evidence-based practice improve patient outcomes? A systematic review. Nurse Educ Today.

[13] Dawes M, Summerskill W, Glasziou P, Cartabellotta A, Martin J, Hopayian K, Porzsolt F, Burls A, Osborne J (2005). Sicily statement on evidence-based practice. BMC Med Educ.

[14] Kim JS, Gu MO, Chang H (2019). Effects of an evidence-based practice education program using multifaceted interventions: a quasi-experimental study with undergraduate nursing students. BMC Med Educ.

[15] Kaper NM, Swennen MH, van Wijk AJ, Kalkman CJ, van Rheenen N, van der Graaf Y, van der Heijden GJ (2015). The "evidence-based practice inventory": reliability and validity was demonstrated for a novel instrument to identify barriers and facilitators for evidence based practice in health care. J Clin Epidemiol.

[16] Fernandez-Dominguez JC, de Pedro-Gomez JE, Morales-Asencio JM, Bennasar-Veny M, Sastre-Fullana P, Sese-Abad A (2017). Health sciences-evidence based practice questionnaire (HS-EBP) for measuring transprofessional evidence-based practice: creation, development and psychometric validation. PLoS One.

[17] Fernandez-Dominguez JC, Sese-Abad A, Morales-Asencio JM, Sastre-Fullana P, Pol-Castaneda S, de Pedro-Gomez JE (2016). Content validity of a health science evidence-based practice questionnaire (HS-EBP) with a web-based modified Delphi approach. Int J Qual Health Care.

[18] Shaneyfelt T, Baum KD, Bell D, Feldstein D, Houston TK, Kaatz S, Whelan C, Green M (2006). Instruments for evaluating education in evidence-based practice: a systematic review. JAMA.

[19] Kate G, Peter A, Anne L, Jeff B, Jo C, Sally K, Elaine MN (2007). Factors influencing the development of evidence-based practice: a research tool. J Adv Nurs.

[20] Shuman CJ, Ploutz-Snyder RJ, Titler MG (2018). Development and testing of the nurse manager EBP competency scale. West J Nurs Res.

[21] Saunders H, Stevens KR, Vehvilainen-Julkunen K (2016). Nurses' readiness for evidence-based practice at Finnish university hospitals: a national survey. J Adv Nurs.

[22] Chang AM, Crowe L (2011). Validation of scales measuring self-efficacy and outcome expectancy in evidence-based practice. Worldviews Evid-Based Nurs.

[23] Tucker SJ, Olson ME, Frusti DK (2009). Evidence-based practice self-efficacy scale preliminary reliability and validity. Clin Nurse Spec.

[24] Connor L, Paul F, McCabe M, Ziniel S (2017). Measuring Nurses' value, implementation, and knowledge of evidence-based practice: further psychometric testing of the quick-EBP-VIK survey. Worldviews Evid-Based Nurs.

[25] Upton D, Upton P (2006). Development of an evidence-based practice questionnaire for nurses. J Adv Nurs.

[26] Melnyk BM, Fineout Overholt E, Mays MZ (2008). The evidence-based practice beliefs and implementation scales: psychometric properties of two new instruments. Worldviews Evid-Based Nurs.

[27] Leung K, Trevena L, Waters D (2014). Systematic review of instruments for measuring nurses' knowledge, skills and attitudes for evidence-based practice. J Adv Nurs.

[28] McEvoy MP, Williams MT, Olds TS (2010). Development and psychometric testing of a trans-professional evidence-based practice profile questionnaire. Med Teach.

[29] Panczyk M, Belowska J, Zarzeka A, Samolinski L, Zmuda-Trzebiatowska H, Gotlib J (2017). Validation study of the polish version of the evidence-based practice profile questionnaire. BMC Med Educ.

[30] Titlestad KB, Snibsoer AK, Stromme H, Nortvedt MW, Graverholt B, Espehaug B (2017). Translation, cross-cultural adaption and measurement properties of the evidence-based practice profile. BMC Res Notes.

[31] Yang RM, Tang SY (2010). The preliminary revision and application of the evidence-based practice questionnaire and the developing evidence-based practice questionnaire.

[32] Zhou F, Hao Y, Guo H, Liu H (2016). Attitude, knowledge, and practice on evidence-based nursing among registered nurses in traditional Chinese medicine hospitals: a multiple center cross-sectional survey in China. Evid Based Complement Alternat Med.

[33] Beaton DE, Bombardier C, Guillemin F, Ferraz MB (2000). Guidelines for the process of cross-cultural adaptation of self-report measures. Spine (Phila Pa 1976).

[34] Polit DF, Beck CT (2006). The content validity index: are you sure you know what's being reported? Critique and recommendations. Res Nurs Health.

[35] Sousa VD, Rojjanasrirat W (2011). Translation, adaptation and validation of instruments or scales for use in cross-cultural health care research: a clear and user-friendly guideline. J Eval Clin Pract.

[36] Gorsuch RL (1983). Factor analysis.

[37] Marsh HW, Hau KT, Balla JR, Grayson D (1998). Is more ever too much? The number of indicators per factor in confirmatory factor analysis. Multivariate Behav Res.

[38] Mosier CI, McQuitty JV (1940). Methods of item validation and abacs for item-test correlation and critical ratio of upper-lower difference. Psychometrica.

[39] Field A (2013). Discovering statistics using IBM SPSS statistics.

[40] Cronbach L (1951). Coefficient alpha and the internal structure of tests. Psychometrica.

[41] Hair JF, Black WC, Babin BJ, Anderson RE (2014). Multivariate Data Analysis.

[42] Bollen KA. Structural Equations with Latent Variables. New York: Wiley; 1989.

[43] Bentler PM (1990). Comparative fit indexes in structural models. Psychol Bull.

[44] MacCallum RC, Browne MW, Sugawara HM (1996). Power analysis and determination of sample size for covariance structure modeling. Psychol Methods.

[45] Santos JRA (1999). Cronbach's alpha: a tool for assessing the reliability of scales. J Ext.

[46] Raykov T (1997). Estimation of composite reliability for congeneric measures. Appl Psych Meas.

[47] Andresen E (2000). Criteria for assessing the tools of disability outcomes research. Arch Phys Med Rehabil.

[48] Hawthorne G, Elliott P (2005). Imputing cross-sectional missing data: comparison of common techniques. Aust N Z J Psychiatry.

[49] Kaiser HF (1974). An index of factorial simplicity. Psychometrica.

[50] Saunders H, Vehvilainen-Julkunen K (2017). Nurses' evidence-based practice beliefs and the role of evidence-based practice mentors at university hospitals in Finland. Worldviews Evid-Based Nurs.

[51] Zabaleta-del-Olmo E, Subirana-Casacuberta M, Ara-Perez A, Escuredo-Rodriguez B, Rios-Rodriguez MA, Carres-Esteve L, Jodar-Sola G, Lejardi-Estevez Y, Nuix-Baque N, Aguas-Lluch A (2016). Developing evidence-based practice questionnaire for community health nurses: reliability and validity of a Spanish adaptation. J Clin Nurs.

[52] Peterson RA, Kim Y (2013). On the relationship between coefficient alpha and composite reliability. J Appl Psychol.

[53] Connor L (2018). Pediatric Nurses' knowledge, values, and implementation of evidence-based practice and use of two patient safety goals. J Pediatr Nurs.

