# Supplementary material for: Development and validation of the Chinese version of the evidence-based practice profile questionnaire (EBP2Q)
Source: BMC Med Educ. 2020 Aug 24;20:280. doi: 10.1186/s12909-020-02189-z (PMC7445933; doi:10.1186/s12909-020-02189-z)
Supplement: Supplementary file 1 — Additional file 1. Characteristics of the Experts in Content Validity of the Questionnaire (n = 6). [file 12909_2020_2189_MOESM1_ESM.docx]

| **Additional File 1.** Characteristics of the Experts in Content Validity of the Questionnaire (n = 6) | | | | | | | |
| --- | --- | --- | --- | --- | --- | --- | --- |
| Expert | Gender | Age | Home town | Education Background | Professional Title | Specialty | Years of working |
| A | Female | 52 | Hunan | PhD in Nursing from Central South University | Professor, Tutor of Master in Nursing at Hunan Normal University, Member of CNA Nursing Education Expert Bank | Nursing Education, Nursing Research | 33 |
| B | Female | 45 | Jiangxi | MSN from First Military Medical University | Associate Professor, Tutor of Master in Nursing at Southern Medical University, Editor of "Chinese Journal of Practical Nursing" | Nursing Education, Nursing Research | 22 |
| C | Female | 36 | Hunan | MSN from England | Clinical Nurse in the Nanfang Hospital of Southern Medical University, Intermediate Title, Member of JBI global EBN China south cooperative center | EBN, Clinical Nursing | 13 |
| D | Female | 34 | Guangdong | MSN from England | Clinical nurse in the Fifth Affiliated Hospital of Guangzhou Medical University, Intermediate Title, Member of Guangdong EBN committee | Clinical Nursing, EBN | 8 |
| E | Female | 32 | Hunan | MSN from England and Southern Medical University | Associate senior editor of the "Journal of Nursing (China)", Deputy director of JBI global EBN China south cooperative center | EBN, Nursing Research | 13 |
| F | Male | 30 | Hubei | MSN from Peking University | Clinical Nurse in Yichang Central People’s Hospital in Hubei, Intermediate Title, Editor of "Frontiers in Bioscience - Landmark" | Clinical Nursing, Nursing Research | 5 |
| **Note:** EBN, Evidence-Based Nursing; JBI, Joanna Briggs Institute; MSN, Master of Science in Nursing; CNA, Chinese Nursing Association | | | | | | |  |
