# Supplementary material for: Development and validation of the Chinese version of the evidence-based practice profile questionnaire (EBP2Q)
Source: BMC Med Educ. 2020 Aug 24;20:280. doi: 10.1186/s12909-020-02189-z (PMC7445933; doi:10.1186/s12909-020-02189-z)
Supplement: Supplementary file 2 — Additional file 2. Item level content validity index (I-CVI) of the pre-final version of EBP2Q (n = 6). [file 12909_2020_2189_MOESM2_ESM.docx]

| **Additional file 2.** Item level content validity index (I-CVI) of the pre-final version of EBP^2^Q (n = 6) | | | | | | |
| --- | --- | --- | --- | --- | --- | --- |
| Item | Number of experts scored 3/4 of the item | | | I-CVI | | |
|  |  |  |  |  |  |  |
|  | Relevance | Clarity | Equivalence | Relevance | Clarity | Equivalence |
| 1 | 6 | 6 | 6 | 1 | 1 | 1 |
| 2 | 6 | 6 | 6 | 1 | 1 | 1 |
| 3 | 6 | 5 | 5 | 1 | 0.83 | 0.83 |
| 4 | 6 | 6 | 6 | 1 | 1 | 1 |
| 5 | 6 | 6 | 6 | 1 | 1 | 1 |
| 6 | 6 | 6 | 6 | 1 | 1 | 1 |
| 7 | 6 | 6 | 6 | 1 | 1 | 1 |
| 8 | 6 | 6 | 6 | 1 | 1 | 1 |
| 9 | 6 | 6 | 6 | 1 | 1 | 1 |
| 10 | 6 | 5 | 5 | 1 | 0.83 | 0.83 |
| 11 | 6 | 6 | 6 | 1 | 1 | 1 |
| 12 | 6 | 5 | 5 | 1 | 0.83 | 0.83 |
| 13 | 6 | 6 | 6 | 1 | 1 | 1 |
| 14 | 6 | 6 | 6 | 1 | 1 | 1 |
| 15 | 5 | 5 | 5 | 0.83 | 0.83 | 0.83 |
| 16 | 6 | 6 | 6 | 1 | 1 | 1 |
| 17 | 5 | 5 | 6 | 0.83 | 0.83 | 1 |
| 18 | 6 | 6 | 6 | 1 | 1 | 1 |
| 19 | 6 | 6 | 6 | 1 | 1 | 1 |
| 20 | 6 | 6 | 6 | 1 | 1 | 1 |
| 21 | 6 | 6 | 6 | 1 | 1 | 1 |
| 22 | 6 | 6 | 6 | 1 | 1 | 1 |
| 23 | 6 | 6 | 6 | 1 | 1 | 1 |
| 24 | 6 | 6 | 6 | 1 | 1 | 1 |
| 25 | 6 | 6 | 6 | 1 | 1 | 1 |
| 26 | 6 | 6 | 6 | 1 | 1 | 1 |
| 27 | 6 | 6 | 6 | 1 | 1 | 1 |
| 28 | 6 | 6 | 6 | 1 | 1 | 1 |
| 29 | 5 | 6 | 6 | 0.83 | 1 | 1 |
| 30 | 6 | 6 | 6 | 1 | 1 | 1 |
| 31 | 5 | 6 | 6 | 0.83 | 1 | 1 |
| 32 | 6 | 6 | 6 | 1 | 1 | 1 |
| 33 | 6 | 6 | 5 | 1 | 1 | 0.83 |
| 34 | 5 | 5 | 6 | 0.83 | 0.83 | 1 |
| 35 | 6 | 6 | 6 | 1 | 1 | 1 |
| 36 | 6 | 6 | 6 | 1 | 1 | 1 |
| 37 | 6 | 6 | 6 | 1 | 1 | 1 |
| 38 | 6 | 6 | 6 | 1 | 1 | 1 |
| 39 | 6 | 6 | 6 | 1 | 1 | 1 |
| 40 | 6 | 6 | 6 | 1 | 1 | 1 |
| 41 | 6 | 6 | 6 | 1 | 1 | 1 |
| 42 | 6 | 5 | 5 | 1 | 0.83 | 0.83 |
| 43 | 6 | 6 | 6 | 1 | 1 | 1 |
| 44 | 6 | 6 | 6 | 1 | 1 | 1 |
| 45 | 6 | 6 | 6 | 1 | 1 | 1 |
| 46 | 6 | 6 | 6 | 1 | 1 | 1 |
| 47 | 6 | 6 | 6 | 1 | 1 | 1 |
| 48 | 6 | 6 | 6 | 1 | 1 | 1 |
| 49 | 6 | 6 | 6 | 1 | 1 | 1 |
| 50 | 6 | 6 | 6 | 1 | 1 | 1 |
| 51 | 6 | 6 | 6 | 1 | 1 | 1 |
| 52 | 6 | 6 | 6 | 1 | 1 | 1 |
| 53 | 6 | 6 | 6 | 1 | 1 | 1 |
| 54 | 6 | 6 | 6 | 1 | 1 | 1 |
| 55 | 6 | 6 | 6 | 1 | 1 | 1 |
| 56 | 6 | 6 | 6 | 1 | 1 | 1 |
| 57 | 6 | 6 | 6 | 1 | 1 | 1 |
| 58 | 6 | 6 | 6 | 1 | 1 | 1 |
| **Note:** Scale level content validity (S-CVI): Relevance-0.985, Clarity-0.979, Equivalence-0.982 | | | | | | |
