# Supplementary material for: Development and validation of the Chinese version of the evidence-based practice profile questionnaire (EBP2Q)
Source: BMC Med Educ. 2020 Aug 24;20:280. doi: 10.1186/s12909-020-02189-z (PMC7445933; doi:10.1186/s12909-020-02189-z)
Supplement: Supplementary file 3 — Additional file 3. Item Analysis of the 58-item Evidence-Based Practice Profile Questionnaire (n = 303). [file 12909_2020_2189_MOESM3_ESM.docx]

| **Additional file 3**. Item Analysis of the 58-item Evidence-Based Practice Profile Questionnaire  (n = 303) | | | | |
| --- | --- | --- | --- | --- |
| Items | CR values | Item-total correlation | α if item deleted | Cronbach’s α |
|  |  |  |  |  |
| Relevance |  |  |  | 0.933 |
| 1 | 11.458** | 0.675** | 0.927 |  |
| 2 | 10.066** | 0.635** | 0.928 |  |
| 3 | 8.805** | 0.568** | 0.930 |  |
| 4 | 10.502** | 0.622** | 0.930 |  |
| 5 | 9.320** | 0.580** | 0.926 |  |
| 6 | 9.734** | 0.572** | 0.925 |  |
| 7 | 10.394** | 0.572** | 0.925 |  |
| 8 | 10.606** | 0.578** | 0.926 |  |
| 9 | 10.860** | 0.538** | 0.927 |  |
| 10 | 9.286** | 0.534** | 0.928 |  |
| 11 | 8.844** | 0.505** | 0.928 |  |
| 12 | 9.805** | 0.515** | 0.928 |  |
| 13 | 8.912** | 0.497** | 0.929 |  |
| 14 | 8.368** | 0.453** | 0.930 |  |
| Sympathy |  |  |  | 0.845 |
| 15 | 2.783* | 0.165** | 0.836 |  |
| 16 | 6.002** | 0.389** | 0.812 |  |
| 17 | 4.264** | 0.310** | 0.822 |  |
| 18 | 4.830** | 0.364** | 0.818 |  |
| 19 | 3.771** | 0.262** | 0.856 |  |
| 20 | 4.888** | 0.346** | 0.810 |  |
| 21 | 6.872** | 0.421** | 0.808 |  |
| Terminology |  |  |  | 0.933 |
| 22 | 8.125** | 0.436** | 0.931 |  |
| 23 | 8.528** | 0.435** | 0.932 |  |
| 24 | 10.853** | 0.590** | 0.930 |  |
| 25 | 8.731** | 0.506** | 0.931 |  |
| 26 | 13.615** | 0.670** | 0.931 |  |
| 27 | 12.808** | 0.597** | 0.930 |  |
| 28 | 13.623** | 0.694** | 0.930 |  |
| 29 | 14.709** | 0.699** | 0.929 |  |
| 30 | 13.234** | 0.698** | 0.929 |  |
| 31 | 11.160** | 0.594** | 0.929 |  |
| 32 | 14.781** | 0.670** | 0.929 |  |
| 33 | 7.484** | 0.425** | 0.932 |  |
| 34 | 9.926** | 0.529** | 0.931 |  |
| 35 | 14.700** | 0.659** | 0.930 |  |
| 36 | 12.106** | 0.650** | 0.928 |  |
| 37 | 11.983** | 0.647** | 0.928 |  |
| 38 | 12.043** | 0.602** | 0.928 |  |
| Practice |  |  |  | 0.923 |
| 39 | 11.984** | 0.608** | 0.921 |  |
| 40 | 14.824** | 0.698** | 0.911 |  |
| 41 | 14.849** | 0.726** | 0.910 |  |
| 42 | 12.909** | 0.662** | 0.912 |  |
| 43 | 14.246** | 0.708** | 0.909 |  |
| 44 | 9.111** | 0.526** | 0.922 |  |
| 45 | 11.742** | 0.662** | 0.911 |  |
| 46 | 12.825** | 0.673** | 0.911 |  |
| 47 | 12.146** | 0.657** | 0.918 |  |
| Confidence |  |  |  | 0.947 |
| 48 | 12.087** | 0.649** | 0.944 |  |
| 49 | 9.276** | 0.523** | 0.947 |  |
| 50 | 9.626** | 0.545** | 0.947 |  |
| 51 | 12.077** | 0.661** | 0.942 |  |
| 52 | 12.613** | 0.659** | 0.941 |  |
| 53 | 14.584** | 0.716** | 0.941 |  |
| 54 | 14.782** | 0.715** | 0.940 |  |
| 55 | 13.371** | 0.706** | 0.940 |  |
| 56 | 13.260** | 0.683** | 0.940 |  |
| 57 | 12.643** | 0.655** | 0.940 |  |
| 58 | 12.840** | 0.680** | 0.941 |  |
| **p<0.001; *p<0.01 | | | |  |
