# Supplementary material for: Development and validation of the Chinese version of the evidence-based practice profile questionnaire (EBP2Q)
Source: BMC Med Educ. 2020 Aug 24;20:280. doi: 10.1186/s12909-020-02189-z (PMC7445933; doi:10.1186/s12909-020-02189-z)
Supplement: Supplementary file 4 — Additional file 4. Exploratory factor analysis of the 54-item Chinese Evidence-Based Practice Profile Questionnaire (n = 303). [file 12909_2020_2189_MOESM4_ESM.docx]

| **Additional file 4.** Exploratory factor analysis of the 54-item Chinese Evidence-Based Practice Profile Questionnaire (n = 303) | | | | | | | | |
| --- | --- | --- | --- | --- | --- | --- | --- | --- |
|  | Factors | | | | | | | |
| Items | 1 | 2 | 3 | 4 | 5 | 6 | 7 | 8 |
| 57 | 0.831 |  |  |  |  |  |  |  |
| 56 | 0.825 |  |  |  |  |  |  |  |
| 55 | 0.816 |  |  |  |  |  |  |  |
| 58 | 0.790 |  |  |  |  |  |  |  |
| 54 | 0.772 |  |  |  |  |  |  |  |
| 52 | 0.755 |  |  |  |  |  |  |  |
| 51 | 0.726 |  |  |  |  |  |  |  |
| 53 | 0.714 |  |  |  |  |  |  |  |
| 48 | 0.671 |  |  |  |  |  |  |  |
| 43 |  | 0.744 |  |  |  |  |  |  |
| 42 |  | 0.730 |  |  |  |  |  |  |
| 45 |  | 0.719 |  |  |  |  |  |  |
| 40 |  | 0.714 |  |  |  |  |  |  |
| 41 |  | 0.677 |  |  |  |  |  |  |
| 46 |  | 0.639 |  |  |  |  |  |  |
| 44 |  | 0.636 |  |  |  |  |  |  |
| 39 |  | 0.552 |  |  |  |  |  |  |
| 14 |  |  | 0.782 |  |  |  |  |  |
| 11 |  |  | 0.764 |  |  |  |  |  |
| 10 |  |  | 0.759 |  |  |  |  |  |
| 13 |  |  | 0.745 |  |  |  |  |  |
| 9 |  |  | 0.706 |  |  |  |  |  |
| 12 |  |  | 0.704 |  |  |  |  |  |
| 31 |  |  |  | 0.731 |  |  |  |  |
| 38 |  |  |  | 0.728 |  |  |  |  |
| 22 |  |  |  | 0.708 |  |  |  |  |
| 33 |  |  |  | 0.690 |  |  |  |  |
| 37 |  |  |  | 0.664 |  |  |  |  |
| 27 |  |  |  | 0.600 |  |  |  |  |
| 6 |  |  |  |  | 0.821 |  |  |  |
| 5 |  |  |  |  | 0.797 |  |  |  |
| 7 |  |  |  |  | 0.791 |  |  |  |
| 8 |  |  |  |  | 0.749 |  |  |  |
| 20 |  |  |  |  |  | 0.801 |  |  |
| 21 |  |  |  |  |  | 0.777 |  |  |
| 16 |  |  |  |  |  | 0.776 |  |  |
| 17 |  |  |  |  |  | 0.746 |  |  |
| 18 |  |  |  |  |  | 0.728 |  |  |
| 2 |  |  |  |  |  |  | 0.804 |  |
| 3 |  |  |  |  |  |  | 0.800 |  |
| 4 |  |  |  |  |  |  | 0.799 |  |
| 1 |  |  |  |  |  |  | 0.611 |  |
| 28 |  |  |  |  |  |  |  | 0.721 |
| 29 |  |  |  |  |  |  |  | 0.714 |
| 26 |  |  |  |  |  |  |  | 0.596 |
| Eigenvalues | 16.518 | 4.492 | 2.558 | 2.411 | 1.984 | 1.550 | 1.332 | 1.111 |
| variance (%) | 36.707 | 9.983 | 5.685 | 5.358 | 4.408 | 3.444 | 2.960 | 2.469 |
| Cumulative (%) | 36.707 | 46.69 | 52.374 | 57.733 | 62.141 | 65.586 | 68.546 | 71.014 |
