# Supplementary material for: Development and validation of the Chinese version of the evidence-based practice profile questionnaire (EBP2Q)
Source: BMC Med Educ. 2020 Aug 24;20:280. doi: 10.1186/s12909-020-02189-z (PMC7445933; doi:10.1186/s12909-020-02189-z)
Supplement: Supplementary file 5 — Additional file 5. Item Analysis of the Revised 45-item Chinese Evidence-Based Practice Profile Questionnaire (n = 303). [file 12909_2020_2189_MOESM5_ESM.docx]

| **Additional file 5.** Item Analysis of the Revised 45-item Chinese Evidence-Based Practice Profile Questionnaire (n = 303) | | | | | |
| --- | --- | --- | --- | --- | --- |
| Itemsa | Mean | SD | Item-total correlation | α if item deleted | Cronbach’s α |
| Basic understanding |  |  |  |  | 0.912 |
| 1 | 3.19 | 0.971 | 0.696** | 0.907 |  |
| 2 | 2.96 | 0.947 | 0.654** | 0.867 |  |
| 3 | 2.99 | 0.958 | 0.590** | 0.892 |  |
| 4 | 2.92 | 0.970 | 0.634** | 0.877 |  |
| Intention |  |  |  |  | 0.945 |
| 5 | 3.63 | 0.893 | 0.621** | 0.931 |  |
| 6 | 3.68 | 0.903 | 0.620** | 0.918 |  |
| 7 | 3.77 | 0.905 | 0.620** | 0.925 |  |
| 8 | 3.63 | 0.907 | 0.623** | 0.939 |  |
| Attitude |  |  |  |  | 0.904 |
| 9 | 4.05 | 0.740 | 0.584** | 0.885 |  |
| 10 | 4.13 | 0.723 | 0.571** | 0.882 |  |
| 11 | 4.11 | 0.702 | 0.543** | 0.883 |  |
| 12 | 4.05 | 0.691 | 0.554** | 0.888 |  |
| 13 | 3.85 | 0.739 | 0.533** | 0.892 |  |
| 14 | 3.95 | 0.751 | 0.487** | 0.892 |  |
| Sympathy |  |  |  |  | 0.851 |
| 16 | 3.35 | 0.901 | 0.401** | 0.818 |  |
| 17 | 3.15 | 0.835 | 0.321** | 0.835 |  |
| 18 | 3.12 | 1.005 | 0.374** | 0.833 |  |
| 20 | 3.17 | 0.850 | 0.367** | 0.808 |  |
| 21 | 3.37 | 0.881 | 0.445** | 0.808 |  |
| Clinical related terms |  |  |  |  | 0.847 |
| 22 | 2.77 | 1.022 | 0.374** | 0.841 |  |
| 27 | 3.17 | 1.100 | 0.553** | 0.834 |  |
| 31 | 2.18 | 0.951 | 0.560** | 0.809 |  |
| 33 | 2.38 | 0.985 | 0.386** | 0.831 |  |
| 37 | 2.36 | 1.073 | 0.607** | 0.810 |  |
| 38 | 2.35 | 1.044 | 0.571** | 0.800 |  |
| EBP related terms |  |  |  |  | 0.867 |
| 26 | 2.5 | 1.091 | 0.663** | 0.912 |  |
| 28 | 2.32 | 1.142 | 0.682** | 0.759 |  |
| 29 | 2.25 | 1.111 | 0.682** | 0.751 |  |
| Practice |  |  |  |  | 0.918 |
| 39 | 2.13 | 1.037 | 0.614** | 0.917 |  |
| 40 | 2.57 | 0.970 | 0.717** | 0.903 |  |
| 41 | 2.71 | 1.045 | 0.743** | 0.902 |  |
| 42 | 2.32 | 0.980 | 0.673** | 0.905 |  |
| 43 | 2.54 | 0.982 | 0.718** | 0.902 |  |
| 44 | 2.7 | 1.052 | 0.535** | 0.918 |  |
| 45 | 2.77 | 1.055 | 0.678** | 0.904 |  |
| 46 | 2.43 | 1.017 | 0.683** | 0.907 |  |
| Confidence |  |  |  |  | 0.949 |
| 48 | 1.86 | 0.911 | 0.647** | 0.947 |  |
| 51 | 2.33 | 0.886 | 0.641** | 0.945 |  |
| 52 | 2.45 | 0.889 | 0.636** | 0.944 |  |
| 53 | 2.3 | 0.992 | 0.714** | 0.943 |  |
| 54 | 2.22 | 0.960 | 0.716** | 0.941 |  |
| 55 | 1.95 | 0.881 | 0.695** | 0.940 |  |
| 56 | 2.06 | 0.895 | 0.679** | 0.940 |  |
| 57 | 2.18 | 0.933 | 0.655** | 0.941 |  |
| 58 | 2.17 | 0.920 | 0.691** | 0.941 |  |
| Note: EBP, Evidence-Based Practice; aItem numbers related to the original Evidence-Based Practice Profile Questionnaire | | | | | |
| **p<0.001 | | | | | |
